# Supplementary material for: Does pruning affect the structural and ecological productivity of Juniper woodlands in the eastern Hindu Kush?
Source: PeerJ. 2025 Jun 17;13:e19184. doi: 10.7717/peerj.19184 (PMC12180449; doi:10.7717/peerj.19184)
Supplement: Supplemental Information 1 — Note: BA, basal area; IVI, Importance value index; *, particular species was absent. [file peerj-13-19184-s001.docx]

| **Stands** | **Species** | **Origin** | **Family** | **Density ha^-1^** | **BA m^2^ ha^-1^** | **IVI** |
| --- | --- | --- | --- | --- | --- | --- |
| MPS | *Juniperus semiglobosa* | Native | Cupressaceae | 86 | 9.9 | 33 |
|  | *Juniperus excelsa* | Native | Cupressaceae | 57 | 4.4 | 23 |
|  | *Elaeagnus angustifolia* | Native | Elaeagnaceae | 73 | 3.11 | 19.23 |
|  | *Salix alba* | Native | Salicaceae | 55 | 1.99 | 16.45 |
|  | *Populus alba* | Native | Salicaceae | 15 | 0.56 | 3.6 |
|  | *Hippophae rhamnoides* | Native | Elaeagnaceae | 15 | 0.49 | 4.7 |
| IPS | *Juniperus semiglobosa* | Native | Cupressaceae | 90 | 3.9 | 36 |
|  | *Juniperus excelsa* | Native | Cupressaceae | 49 | 2.4 | 17 |
|  | *Elaeagnus angustifolia* | Native | Elaeagnaceae | 57 | 2.35 | 18.5 |
|  | *Salix alba* | Native | Salicaceae | 44 | 1.51 | 15.21 |
|  | *Populus alba* | Native | Salicaceae | 14 | 0.93 | 8.61 |
|  | *Hippophae rhamnoides* | Native | Elaeagnaceae | 11 | 0.27 | 3.75 |
| NPS | *Juniperus semiglobosa* | Native | Cupressaceae | 78 | 16 | 31 |
|  | *Juniperus excelsa* | Native | Cupressaceae | 73 | 5.44 | 29 |
|  | *Elaeagnus angustifolia* | Native | Elaeagnaceae | 43 | 4.1 | 14.3 |
|  | *Salix alba* | Native | Salicaceae | 36 | 0.86 | 13.09 |
|  | *Populus alba* | * | * | * | * | * |
|  | *Hippophae rhamnoides* | Native | Elaeagnaceae | 18 | 0.44 | 2.8 |
